# Tandem repeats derived from centromeric retrotransposons

**DOI:** 10.1186/1471-2164-14-142

**Published:** 2013-03-04

**Authors:** Anupma Sharma, Thomas K Wolfgruber, Gernot G Presting

**Affiliations:** 1 Department of Molecular Biosciences and Bioengineering, University of Hawai‘i at Mānoa, Agricultural Science Building, Rm 218, 1955 East–West Road, Honolulu, HI, 96822, USA

**Keywords:** Centromere, Centromeric retrotransposon, Gene conversion, Homogenization, Recombination, Tandem repeat, Satellite

## Abstract

**Background:**

Tandem repeats are ubiquitous and abundant in higher eukaryotic genomes and constitute, along with transposable elements, much of DNA underlying centromeres and other heterochromatic domains. In maize, centromeric satellite repeat (CentC) and centromeric retrotransposons (CR), a class of Ty3/gypsy retrotransposons, are enriched at centromeres. Some satellite repeats have homology to retrotransposons and several mechanisms have been proposed to explain the expansion, contraction as well as homogenization of tandem repeats. However, the origin and evolution of tandem repeat loci remain largely unknown.

**Results:**

CRM1TR and CRM4TR are novel tandem repeats that we show to be entirely derived from CR elements belonging to two different subfamilies, CRM1 and CRM4. Although these tandem repeats clearly originated in at least two separate events, they are derived from similar regions of their respective parent element, namely the long terminal repeat (LTR) and untranslated region (UTR). The 5^′^ ends of the monomer repeat units of CRM1TR and CRM4TR map to different locations within their respective LTRs, while their 3^′^ ends map to the same relative position within a conserved region of their UTRs. Based on the insertion times of heterologous retrotransposons that have inserted into these tandem repeats, amplification of the repeats is estimated to have begun at least ~4 (CRM1TR) and ~1 (CRM4TR) million years ago. Distinct CRM1TR sequence variants occupy the two CRM1TR loci, indicating that there is little or no movement of repeats between loci, even though they are separated by only ~1.4 Mb.

**Conclusions:**

The discovery of two novel retrotransposon derived tandem repeats supports the conclusions from earlier studies that retrotransposons can give rise to tandem repeats in eukaryotic genomes. Analysis of monomers from two different CRM1TR loci shows that gene conversion is the major cause of sequence variation. We propose that successive intrastrand deletions generated the initial repeat structure, and gene conversions increased the size of each tandem repeat locus.

## Background

Maize centromeres are enriched in the tandem centromeric satellite repeat CentC and centromeric retrotransposons (CR). The centromeric retrotransposons of maize (CRM) are characterized by an integrase that contains a CR motif [1,2], and elements belonging to subfamilies CRM1, CRM2 and CRM3 integrate predominantly at the centromeres of corn [3]. Recombinant elements have been identified within the CRM1 and CRM4 subfamilies [4]. The exact role of the CentC and CRM elements in corn centromeres remains unknown.

Tandem repeats are major constituents of higher eukaryotic genomes and are typically localized to specialized chromosomal regions such as centromeres, telomeres, (see [5] for a review) and heterochromatic knobs [6]. Satellite repeats are thought to play a role in organizing and stabilizing these specialized chromosomal features, which are important for chromosome behavior during cell division [5]. In addition to noncoding satellite repeats, many genes, such as histones and ribosomal RNA genes, are amplified and arrayed in tandem on chromosome arms. These tandemly arrayed genes (TAG) are thought to provide the large quantities of protein or RNA products required for important physiological and biological functions [7,8].

While some satellite repeats are chromosome-specific, others are more broadly distributed. In maize, the 741 nt Cent4 satellite repeat is localized near the centromere of chromosome 4 [9], while the 156 nt centromere specific satellite repeat CentC is present at all centromeres [10]. The 180 bp [11] and 360 bp TR1 tandem knob repeats [12] were detected on all eight maize chromosomes analyzed, although their copy number on different chromosomes varies greatly [13].

Many satellite repeat arrays and TAGs seem to undergo concerted evolution by sequence homogenization. Random homology-dependent unequal crossovers [14], replication slippage followed by unequal crossing over [15], rolling circle replication of extrachromosomal circular DNAs [16-18], segmental duplication [19], and gene conversion [20] have all been proposed to account for the sequence homogeneity, higher order structure, as well as rapid expansion and contraction of tandem arrays.

The origin of novel satellite repeats, however, remains elusive. Tandem repeats with homology to parts of retrotransposons have been identified in several plants, e.g., wheat, rye [21], and potato [22,23]. The Cent4 tandem repeat of maize has homology to telomeric repeat, knob repeat, and the B chromosome centromere [9]. Tandem repeats with homology to intergenic spacer of ribosomal DNA have been described in several plants, including potato [24], common bean [25], tobacco [26], and tomato [27].

Here we report the discovery and characterization of two CR-derived tandem repeats, describe the sequence features shared by them, and propose molecular mechanisms responsible for their generation, amplification and homogenization.

## Results

### CRM1TR – a CRM1-derived tandem repeat

**Figure 1:**
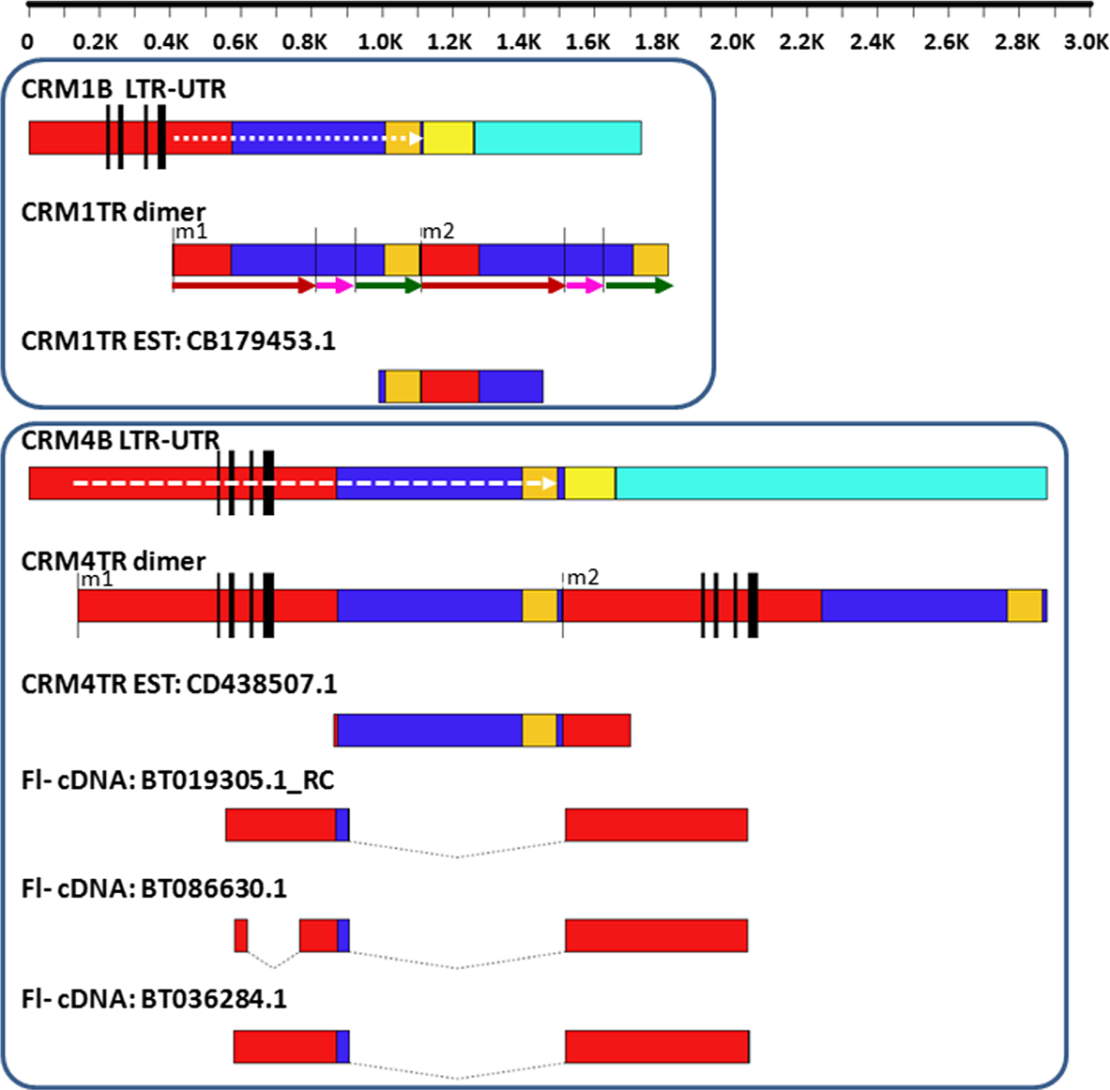
**Schematic representation of CRM1TR and CRM4TR, their parent elements and transcripts.** Schematic illustrations (from top to bottom) of CRM1B LTR-UTR region, CRM1TR dimer (from locus I), CRM1TR derived EST, CRM4B LTR-UTR region, CRM4TR dimer, CRM4TR derived EST, and 3 full length spliced cDNAs derived from CRM4TR repeats. The CRM-derived tandem repeats, CRM1TR and CRM4TR, consist of part of LTR (red) and UTR (dark blue and orange) of the parent elements. Other regions of the parental UTR not included in the tandem repeats are shown in yellow and light blue. Four black vertical bars in the LTR regions of CRM1B and CRM4B mark conserved motifs, i.e. TATA-like box, recombination breakpoint, a C-rich and T-rich region respectively. CRM1TR and CRM4TR monomers both terminate within a region conserved between CRM1B and CRM4 UTRs; the first 100 nucleotides and the last 143 nucleotides of this UTR region are shown in orange and yellow respectively. White dotted arrows within the parental elements indicate the region that formed full length CRM1TR and CRM4TR monomers. Maroon, magenta, and green arrows underneath the CRM1TR dimer indicate subsequences S, IR, and EB (see text for details). Dotted connectors in cDNAs indicate spliced introns. m1 and m2 = monomers 1 and monomer 2. Full length cDNA BT019305.1 was reverse complemented.

Tandem repeats CRM1TR and CRM4TR were discovered serendipitously during BLAST searches of maize genome and cDNA sequences, respectively. CRM1TR is a tandem repeat of maize derived from the maize centromeric retrotransposon subfamily CRM1. Full length CRM1TR monomers have up to 97% sequence similarity to a segment of LTR-UTR of CRM1B retrotransposon (Figure 1). In maize inbred B73, CRM1TR repeat arrays are located at two loci in the pericentromere of chromosome 9 that are separated by ~1.4 Mb: locus I spans coordinates 53,218,796-53,432,137 of RefGen_v2, which is covered by two overlapping BACs (c0393I11 and c0228P17), while locus II lies between 54,849,812 -54,964,918 (BAC c0418G23). All three BACs contain centromeric retrotransposons CRM1 and CRM2, which indicates that these regions were at some point part of the functional centromere. BAC c0393I11 is located at the edge of centromere and part of this BAC and some CRM1TR arrays therein are associated with centromere specific histone H3 variant CENH3 in the maize inbred B73 (data not shown).

CRM1TR arrays are organized into 44 uninterrupted tandem arrays, or islands: 28 islands at locus I and 16 islands at locus II. These islands are separated from each other either by gaps in the physical assembly or by intervening sequences, e.g. LTR retrotransposons. We identified at least 5 full length LTR retrotransposon insertions, dated between 0.54 to 4.09 My (κ = 0.00699 and 0.05319), within CRM1TR arrays at locus I. This indicates that the locus I CRM1TR arrays originated around the time of the allotetraploidization event that resulted in modern day corn [28]. No full-length retrotransposon insertions were detected within locus II CRM1TR arrays, suggesting that locus I is older than locus II, that retrotransposons are more efficiently removed from, or less likely to insert into, locus II.

**Figure 2:**
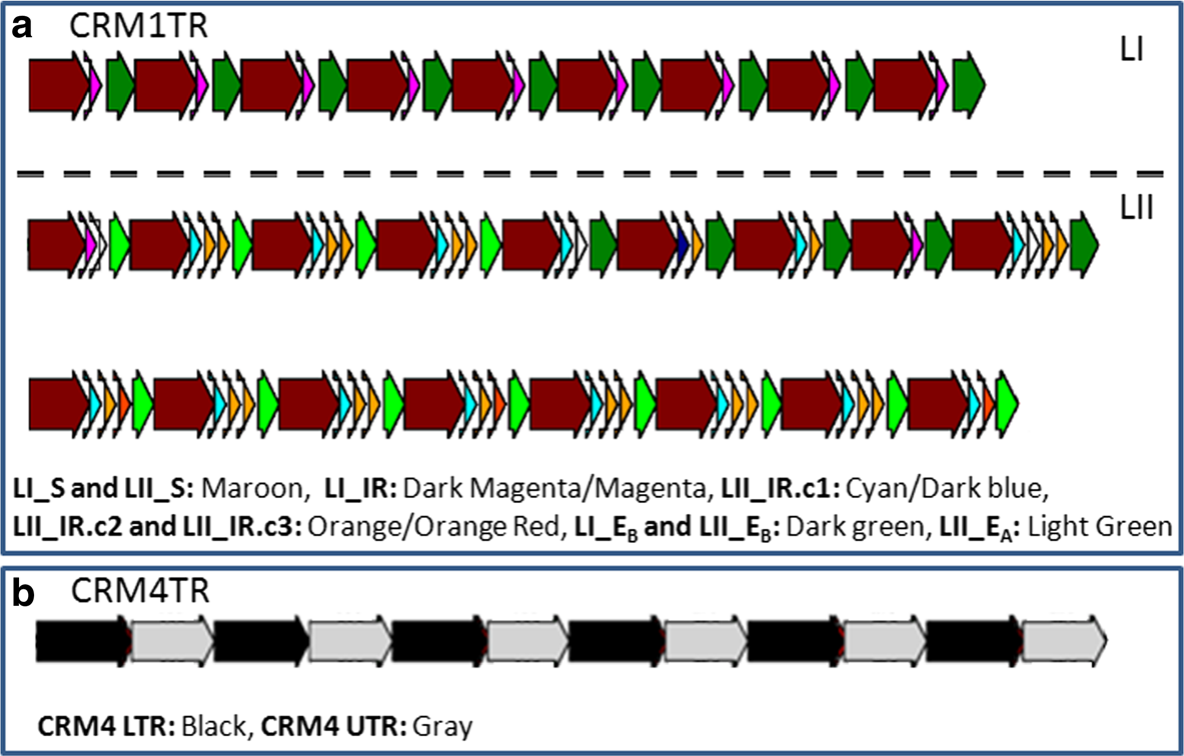
**Schematic representation of CRM1TR and CRM4TR arrays. a**. Domain structure within CRM1TR monomers differs between locus I (L1) and II (LII). Excerpt of small regions from CRM1TR arrays at the two loci show that L1 repeats are homogenous with the structure S-IR-E_B_, while L2 repeats are more heterogeneous with the general structure S-IR*-E_B_/E_A_, where IR* indicates between 1 to 4 copies of internal repeat variants, and E_B_/E_A_ indicates the two end sequence variants. The majority of L2 monomers have three copies of IR (i.e. IR.c1, IR.c2, and IRc3) that can be distinguished based on SNPs. **b**. CRM4TR arrays are homogenous and derived from an LTR-UTR segment of CRM4B. Figures were drawn using JunctionViewer software.

We extracted a total of 187 full-length (fl) CRM1TR monomers (97 from locus I and 90 from locus II). Detailed sequence analysis of these monomers revealed that a typical CRM1TR monomer contains ~402 nt start sequence (S) at its 5^′^ end, one to four tandem copies of an internal repeat (IR) of ~100 nt, and one of two end sequences at its 3^′^ end, the ~185 nt E_B_ or ~138 nt E_A_ (Figure 2a).

Locus I CRM1TR monomers have the general structure ‘S-IR-E_B_’ while those from locus II have the general structure S-IR*-E_A_/E_B_, where IR* indicates between 1 to 4 tandem copies of IR and E_A_/E_B_ indicates either E_A_ or E_B_. Most (66.7%, 60/90) locus II CRM1TR monomers have three tandem copies of IR sequence. Other locus II CRM1TR monomers have either four (6/90), two (22/90), or one (2/90) IR sequence. Consequently, locus II monomers are longer (819 nt average) and more variable in length than locus I monomers (average 696 nt) (Additional file 1). Figure 2a illustrates the structure of CRM1TR monomers as well as their arrangement at locus I and locus II.

### Distinct haplotypes at the two CRM1TR loci suggest local homogenization

To investigate the sequence homogenization within and across CRM1TR arrays, we analyzed diagnostic variant nucleotides (SNPs) within ‘S’ and ‘IR’ sequences from fl-CRM1TR monomers. A set of seven SNPs within ‘S’ distinguishes monomers from locus I and II (Additional file 2). Based on these SNPs, the S sequences from 97 locus I CRM1TR monomers were grouped into 14 haplotypes and those from 90 locus II CRM1TR monomers were grouped into 18 haplotypes. Most S sequences from locus I CRM1TR monomers (76%) are of the haplotype ‘ACGGAGT’ while most of those at locus II are of the haplotype ‘GTCACTC’ (53.9%) or ‘GCCGCTC’ (17.9%) (Additional file 3 and Additional file 4).

No haplotypes of ‘S’ were shared between locus I and II, but, a search of the htgs database of GenBank using the consensus S sequences of locus I and locus II identified the respective parent BAC(s) as the best match followed by the BAC corresponding to the other CRM1TR locus. This suggests that the S haplotypes at the two CRM1TR loci, though distinct, are more closely related to each other than to any full-length CRM element in sequenced maize genome and may even have originated from the same ancestral sequence.

For IR haplotype analysis, we used all locus I CRM1TR monomers but only those locus II monomers with three IR copies. The IR sequences contains a terminal A-rich region that is approximately 38 nt long in locus I monomers and ~25 nt long in locus II monomers (Additional file 2). This A-rich region was not included in SNP analysis because it is highly polymorphic. A set of 7 SNPs distinguished IR sequences from locus I and II. Based on these SNPs, the 97 ‘IR’ sequences from 97 locus I monomers were grouped into 10 haplotypes, whereas the 180 ‘IR’ sequences from 60 ‘IR’ locus II monomers were grouped into 28 haplotypes. Most IR sequences from locus I monomers are of the haplotype CACCCTA (55.5%) or CACCCTC (33%) (Additional file 3 and Additional file 4). Position specific IR haplotypes were detected in locus II monomers with three IR repeats, i.e. haplotype CACCTCT dominates at the first IR, IR.c1 (76.7%, 46/60), the related haplotypes TGACCTA (51.7%, 31/60) or TGACCTC (28.3%, 17/60) dominate at the second IR, IR.c2, and the related haplotypes TGATCTA (63.3%, 38/60) or TGATCTC (18.3%, 11/60) dominate at the third IR, IR.c3 (Additional files 3 and Additional file 4). This position specificity was absent in monomers with one, two or four IRs. For instance, the IR.c3 haplotype TGATCTA is located at the second IR in 12 of 22 monomers with two IRs and at the fourth IR in 3 of 6 monomers with four IRs (data not shown). These data suggests CRM1TR monomers with one, two, or four IRs likely originated from CRM1TR monomers with three IRs via recombination.

The two IR haplotypes present in the majority of locus I monomers are also represented at locus II, albeit only in three locus II monomers, i.e. locus I haplotype CACCCTC also matches the first IR of a one IR and a two IR containing monomer at locus II, and locus I haplotype CACCCTC matches the first IR of a two IR containing monomer at locus II. Nonetheless, all three IR copies from locus II that share haplotype with locus I monomers can be distinguished from locus I monomers based on their shorter A rich region at the termini, which is similar to other locus II monomers. These data may be indicative of a common origin of the two CRM1TR loci or a low level of intrachromosomal gene conversion between the two CRM1TR loci.

Distinct S and IR haplotypes at the two CRM1TR loci suggests that CRM1TR monomers are locally homogenized, and that the two loci followed separate evolutionary trajectories. This is reminiscent of centromeric satellites in Arabidopsis, where repeats derived from the same genomic locus were discovered to be more similar to each other than those from disparate genomic regions [29]. Similarly, analysis of centromeric repeats in human X chromosomes revealed that repeats that are near each other (within ~15 kb) were significantly more similar than those at random loci either on the same or different X chromosomes [30].

### CRM1TR monomers containing three IR originated from existing CRM1s

To assess whether the three IRs present in most locus II CRM1TR monomers formed *de novo* or originated from existing CRM1 elements, we searched the htgs database of GenBank for CRM1 elements with multiple copies of the IR sequence. Most full length CRM1B retrotransposons contain a single IR copy that is similar in sequence to the IR sequence in locus I CRM1TR monomers, but we discovered at least two full length CRM1 copies (refGen_v2 chr4 coordinates 108,400,064-108,393,127 and 107,845,660-107,838,735) that contain three IRs similar in sequence to the three IRs in most locus II CRM1TR monomers. SNPs shared between the IR regions of the two CRM1B elements and the consensus sequence of locus II CRM1TR monomers (Additional file 2 and Additional file 4) suggest that the three IRs in most locus II monomers originated from existing CRM1B elements rather than by *de novo* triplication of IR sequence within locus II CRM1TR monomers.

### CRM1TR monomer end variant E_A_ originated by gene conversion

Locus II CRM1TR monomers contain one of the two end variants, E_A_ or E_B_ (Additional file 2). Sequence comparison to CRM1 A and CRM1 B parent alleles revealed that S, IR and E_B_ sections of CRM1TR are more similar to CRM1B and that only E_A_ is more similar to CRM1A (Table 1). Moreover, none of the full length CRM1B elements in the GenBank htgs database contain the E_A_ sequence, suggesting that the CRM1TR repeat originated from a CRM1B element, and that monomers with E_A_ originated at locus II by replacement of E_B_ with the CRM1A derived E_A_ sequence. These recombinant E_A_ containing monomers account for the majority of (approximately 68%) of CRM1TR monomers at locus II. Conservation of CRM1B derived sequence both upstream and downstream of E_A_ suggests that E_A_ replaced E_B_ via a gene conversion event. SNP analysis indicates that an at least 71 bp (and up to ~200 bp) region of the CRM1TR repeat was converted to a CRM1A type sequence. Sequences upstream (49 nt) and downstream (~85 nt) of this 71 bp region are ~96% and 100% identical to the CRM1A consensus sequence, respectively. In yeast, the gene conversion rate is known to increase with the length of homologous region, and as little as 13 bp of flanking homologous sequence appears to be sufficient for gene conversion [31]. Gene conversion events via the double strand break repair pathway result in short conversion tracts of 1–2 kb [32], though much shorter conversion tracts have been observed for gene conversion events involving repeats [33].

**Table 1:** Sequence similarity between CRM1TR subsequences and CRM1A or CRM1B consensus sequences

	**Maximum bitscore with CRM1_A**	**Maximum bitscore with CRM1_B**
**CRM1TR_I_S**	558	**712**
**CRM1TR_II_S**	553	**717**
**CRM1TR_I_IR**	141	**193**
**CRM1TR_II_IR.c1**	115	**152**
**CRM1TR_ II_IR.c2**	132	**159**
**CRM1TR_ II_IR.c3**	134	**141**
**CRM1TR_I_E**_**B**_	182	**313**
**CRM1TR_ II_E**_**B**_	182	**313**
**CRM1TR_ II_E**_**A**_	**255**	180

Identity of subsequences from CRM1TR monomers at the two CRM1TR loci (I or II) to the two CRM1 subfamily variants CRM1A and CRM1B is shown in bitscores. Note that all subsequences of CRM1TR i.e. S, IR variants (IR, IR.c1, IR.c2, IR.c3) and E_B_ have high homology to CRM1B, except end sequence variant E_A_, which is more similar to CRM1A.

### CRM4TR - a CRM4 derived tandem repeat

CRM4TR is derived from a member of the CRM4 subfamily. Full length CRM4TR monomers share between 95-98% sequence homology to a ~1370 nt LTR-UTR segment (nucleotide positions 138 to 1507) of the full length CRM4B retrotransposon consensus sequence (Figure 1, 2b). CRM4TR repeat arrays are located at a single genomic locus spanning coordinates 47,638,635 to 47,807,586 on chr 6 of RefGen_v2. CRM4TR arrays are located in the pericentromeric region spanned by two overlapping BACs (c0466I13 and c0290C08) that lack CentC as well as other centromere enriched retrotransposons CRM1/2/3 and are located ~1.8 Mb from the functional centromere (data not shown).

CRM4TR arrays are organized into 6 islands separated by either gaps in the physical assembly or intervening sequences, including LTR retrotransposons. We identified two nested insertions of retrotransposon A188 (GenBank accession ZMU11059) in opposite orientation between the first two islands and estimated their insertion times at 0.94 My (LTR edit distance κ = 0.0122) and 0.79 My (κ = 0.0103), suggesting that this CRM4TR array has existed in the maize genome for at least ~1 My and may have formed more recently than CRM1TR locus I.

The 39 fl-CRM4TR monomers from the CRM4TR locus range in length from 1369–1391 nt (average size 1386 nt). CRM4TR consensus sequence contains roughly 13 repetitions of ~18 nt sequence near its 3^′^ end (Additional file 5). Based on shared SNPs, repeats 2, 4, 6, 10, and 12 form group 1, repeats 3, 5, 7 form group 2, and repeats 8, 10, and 12 form group 3. These repeats thus form at least two sets of ~36 nt composite repeat, where set I includes composite pairs 2–3, 4–5, 6–7 and set II includes composite pairs 8–9, 10–11, and 12–13 (Additional file 5).

### CRM1TR versus CRM4TR

CRM1TR and CRM4TR repeats include part of their parent elements’ LTR and UTR, but the relative start sites in the CRM1 and CRM4 LTRs differ. The 5^′^ end of CRM4TR monomers lies ~395 nt upstream of TATA box, and therefore in the U3, and includes several motifs conserved between the CRM1 and CRM4 LTRs, i.e. the TATA box, recombination breakpoint (RB), and the C-rich and T-rich motifs (Additional file 6). In contrast, the 5^′^ end of CRM1TR monomers is located ~180 nt downstream of TATA box in the U5 region and thus lacks all of the sequence motifs listed above.

Remarkably, CRM1TR and CRM4TR monomers terminate at similar locations within their respective UTRs. A 251 nt region of CRM1B UTR is homologous (73% sequence identity) to a 263 nt region of the CRM4 UTR. CRM1TR and CRM4TR monomers terminate within 12 nucleotides of each other, i.e. at position 100 and 113 respectively within this homologous region. The first 99 nucleotides and the terminal 143 nt of the homologous region are 72% and 83% identical between CRM1 and CRM4. The terminal 143 nt of the homologous region also contains a polypurine rich region (Additional file 7). The conserved ~ 250 nt UTR region was not detected in CRM2.

### CRM1TR and CRM4TR repeats are transcribed

A GenBank search revealed one CRM1TR- and one CRM4TR-derived EST spanning the junction between two monomers (Figure 1). In addition, 3 full length cDNAs (Fl-cDNA) originating from CRM4TR repeats were detected in two different cDNA libraries (Additional file 8). These ESTs and cDNA clones were prepared from polyadenylated RNA. All three CRM4TR derived cDNAs had spliced out most of the UTR region and one cDNA had an additional splice in the LTR (Figure 1). The 5^′^ ends of the three CRM4TR derived Fl-cDNAs are located 18, 40, and 43 nt downstream of TATA box in the CRM4TR monomers. No CRM1TR-derived full length cDNAs was detected, which may reflect the lack of a TATA box and other regulatory regions required for transcription by RNA polymerase II. The impact or role, if any, of the inclusion or exclusion of transcription regulatory signal in CRM4TR and CRM1TR on local chromatin organization and/or function is unclear. Although, the role, if any, of non-coding polyadenylated transcripts in plant heterochromatin formation remains largely unknown, a recent study in *Arabidopsis* showed that RNA polymerase II transcription recruits Pol IV and Pol V at heterochromatic loci to promote siRNA biogenesis and siRNA-mediated transcriptional gene silencing [34].

## Discussion

### Repeated creation of tandem repeats from CR elements

The maize genome contains hundreds of full-length centromeric retrotransposons belonging to six different subfamilies (CRM1-CRM6) [35] (Sharma et al. unpublished). Three of these (CRM1-CRM3) have the ability to target their insertion to the functional centromeres as defined by CENH3 [3]. Maize centromeres also contain the tandem repeat CentC, which shares high sequence homology with the CentO repeat found at rice centromeres [36], indicating that this centromeric repeat likely has resided at the centromeres of these two species since they diverged about 50 My. It is not clear how these tandem centromeric repeats arose, but the discovery of tandem repeats derived from CRM1 and CRM4 raises the possibility that CentC and CentO may have been derived from ancient centromeric retrotransposons.

CRM1TR and CRM4TR were created in at least two independent events. The two CRM1TR loci on the other hand seem to have a common origin at least 4 My. The localization of CRM1TR at only two neighboring loci separated by ~1.4 Mb on chr9 suggests that the second CRM1TR locus originated either by retrotransposon insertions and/or other genomic rearrangement that separated the original cluster into two, or by intrachromosomal gene conversion events that transferred some CRM1TR repeat to the second locus possibly by recombination with a CRM1 sequence. Several studies in yeast indicate that intrachromosomal gene conversion events are frequent and may result in long conversion tracts [37-40].

Transposable elements are a major source of tandem repeats although a few cases of acquisition of satellite repeat monomers by transposable elements have also been reported. Roughly one quarter of all minisatellites/satellites in the human genome are derived from transposable elements [41]. The creation of tandem repeats from retrotransposons, including CR elements, has been documented a number of times. For example, at least four of the centromeric repeats in potato are amplified from retrotransposon-related sequences [23] and the 4.7 kb monomer of sobo satellite repeat of wild potato, which spans ~360 kb in the chromosome 7 pericentromere, shares sequence similarity with the LTRs of Sore1 gypsy retrotransposon, satellite repeat, and genomic DNA [22]. In rodent, the 348 bp monomer of satellite repeat RPCS shares sequence identity with the U3 region of the LTR of the Rous sarcoma virus, and contains several sequence elements that are characteristic of retroviral LTRs such as a polypurine tract, CCAAT boxes, a TATA box and putative polyadenylation signals, as well as binding sites for the CCAAT/enhancer-binding protein (C/EBP) and CCAAT proteins related to NF-1 [42,43]. In rye, the satellite repeat family E3900 has sequence derived from a Ty3-gypsy retrotransposon while the D1100 family contains a rearranged MITE element [44]. In wheat and rye, a centromeric repeat with 250 bp repeat unit has 53% amino acid sequence similarity to the Cereba (a CR family retrotransposon) gag gene containing CAA microsatellite [21] and the satellite 1 family of *Xenopus laevis* has homology to a SINEs [45]. Satellite sequences with homology to the 3^′^ UTR regions of plant retrotransposons belonging to the Tat-lineage, which frequently contain variable tandem repeats, have been identified in several plant species [46].

In some cases, partial homology between satellite repeats and retrotransposons has been attributed to acquisition and dispersal of satellite repeat sequence by transposable elements. For example, in *Drosophila*, direct terminal repeats of the functional pDv elements might have been derived from the pvB370 satellite DNA family through insertion of a tandemly repeated 36-bp transcription unit [47]. Similarly, acquisition and subsequent dispersal of part of a TCAST satellite DNA sequence into a retro transposon is proposed to explain the distribution of TCAST element in the vicinity of genes within euchromatin [48].

### Similar regions of CR elements give rise to tandem repeats

We have discovered several parallels between CRM1TR and CRM4TR, which may begin to provide some clues about the transitioning process from autonomous retrotransposon to tandem repeat. First, it is noteworthy that the only CRM subfamilies that gave rise to tandem repeats are those for which recombinant elements have been documented [4]. The CRM1 and CRM4 recombinants were postulated to have arisen from nested insertions of related elements, which suggests that the CR tandem repeats may also have arisen from nested insertions. Second, similar regions of the parental CRM element (i.e. several hundred nucleotides upstream and downstream of the LTR-UTR junction) gave rise to the tandem repeats in each case, indicating perhaps that similar mechanisms were involved in the creation and maintenance of these tandem repeats. Third, all three CRM-derived tandem repeat loci lie in the centromere or pericentromere, regions that are likely subject to large physical forces and possibly frequent chromosome breakage.

### Initiation of tandem repeats by illegitimate recombination

**Figure 3:**
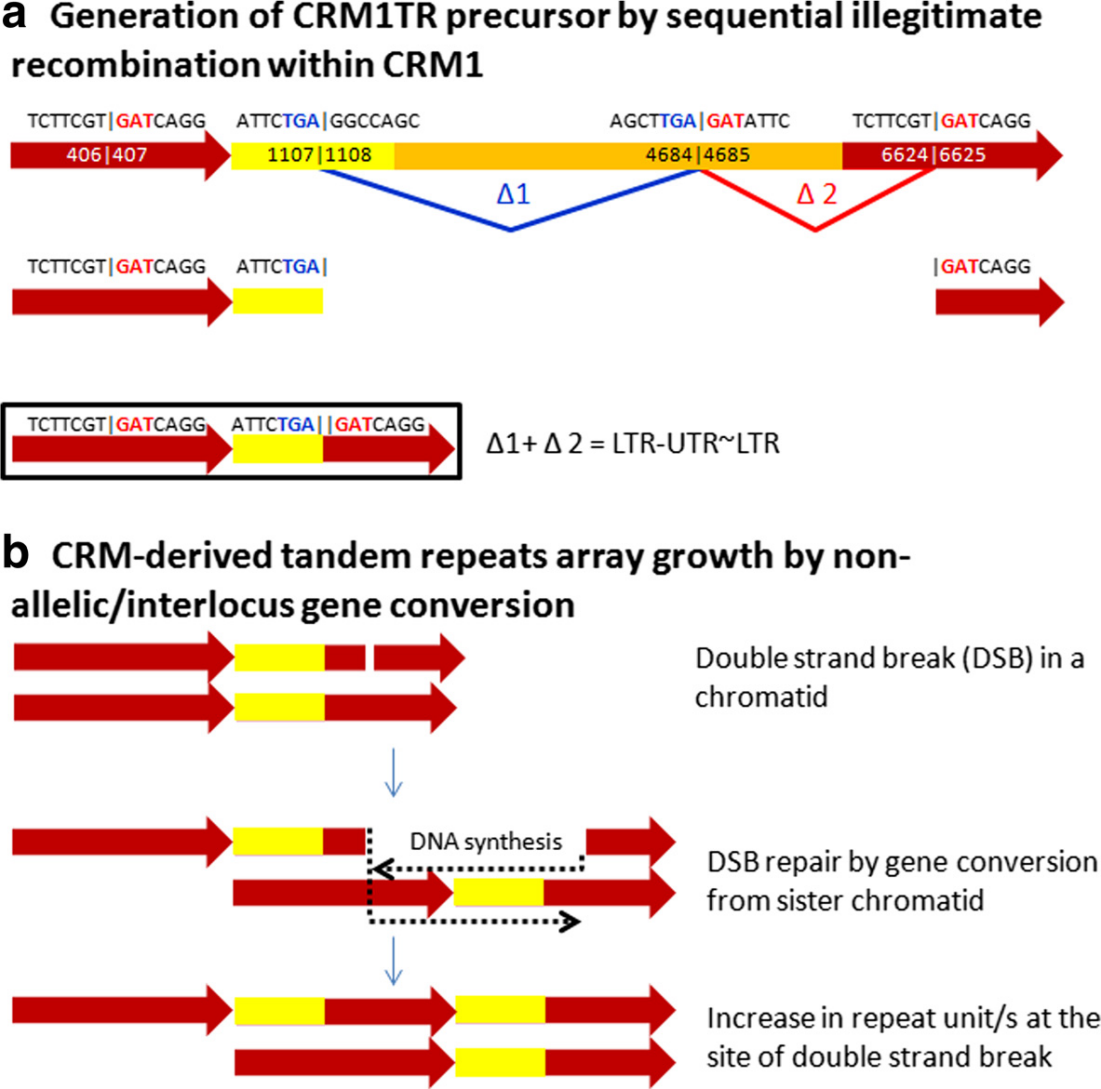
**Model for the generation and growth of CRM-derived tandem repeat arrays. a**. Sequential illegitimate recombinations between short regions of homology, as shown by two deletion events, Δ1 between the trinucleotides TGA shown in blue font and Δ2 between trinucleotides GAT shown in red font above the full length retrotransposon schematic at the top (not to scale), can generate an LTR-UTR~LTR structure (a prerequisite for formation of CRM1TR tandem arrays) containing the characteristic UTR~LTR junction between CRM1TR repeat monomers. **b**. Growth of CRM-derived tandem repeat arrays by non-allelic/interlocus gene conversion. A double strand break in the downstream LTR of LTR-UTR~LTR in one of the chromatids is repaired using the non-allelic LTR of the LTR-UTR~LTR structure on the sister chromatid (bottom) by the standard double strand break (DSB) repair model. The details of the repair (processing of both strands at the site of DSB to generate 3’ overhangs, sister chromatid invasion by the 3’ overhangs, branch migration and resolution of the two Holliday Junctions) are not shown. *Figure not to scale: LTR (red), UTR (yellow), polyprotein (blue).

We propose that CRM-derived tandem arrays arose from a LTR-UTR~LTR structure (where ‘~’ indicates a deletion of an internal region from the UTR to the downstream LTR). The UTR~LTR junction may have formed via intrastrand illegitimate recombination events within a single (as illustrated in Figure 3) or between nested elements. Since the short direct repeats required for formation of UTR~LTR junction via single illegitimate recombination event were not detected in CRM1B and CRM4B consensus sequences (Additional file 9), sequential recombinations would have been required. However, we cannot exclude the possibility that an alternative recombination mechanism akin to an atypical non-homologous end-joining may have generated the splice forming the UTR~LTR junction, as there is evidence that non-homologous end joining can be accompanied by long deletions [32]. DNA sequence likely played a role in defining the CRM1TR and CRM4TR monomers’ 3^′^ termini that map to roughly the same site within a segment of UTR conserved between the CRM1B and CRM4B.

### Amplification of CRM-derived tandem repeats by gene conversion

The fact that CRM-derived repeats are located in or near the centromeres—chromosomal loci where meiotic crossing over is suppressed but gene conversions are frequent [49,50]— suggest that CRM-derived repeats were amplified and homogenized primarily by non-allelic/interlocus gene conversion (Figure 3). The amenability of these tandem repeat loci to gene conversion in general is further evidenced by our discovery of recombinant CRM1TR monomer such as those having replaced their CRM1B type ends with a CRM1A derived sequence. However, we cannot exclude that other mechanisms such as unequal exchange between sister chromatids (during mitosis) [50-52] as well as insertions of the products of rolling circle replication of an extrachromosomal circular DNA [53] may have also contributed to the amplification and homogenization of CRM –derived repeats.

### CRM-derived tandem repeats – a path to centromeric repeats?

The presence of megabase spanning satellite repeats is a hallmark of centromeres, although there is little detectable sequence conservation. For example, in* Oryza brachyantha* the centromeric satellite CentO has been replaced with CentO-F, which has no sequence similarity to CentO, since divergence from *O. sativa* about 10–15 million years ago [54,55]. Centromere specific satellite repeats that span nearly the entire core of six potato centromeres, are composed of long monomers (979 bp to 5.4 kb). Four of these six centromeric repeats are derived from retrotransposon-related sequences [23]. Array sizes of centromere and knob repeats vary substantially in different maize inbreds [56], and maize centromeres have been described as dynamic loci that can shift their position over time away from centromeric satellite repeats [57]. Although the function of centromeric repeats is still unknown, it may be to preferentially bind nucleosomes containing the centromeric histone variant CENH3 [58,59]. CENH3 has been characterized as a protein that evolves rapidly, possibly in concert with centromeric DNA [60]. In vitro nucleosome reconstitution experiments suggest that CentC and the LTR/UTR region of CRM sequences preferentially bind CENH3 nucleosomes (Xie et al. unpublished). Taken together with these facts, the discovery of CRM-derived tandem repeats suggest a mechanism by which the centromeric satellite repeats can be renewed or replaced using rapidly evolving retrotransposon sequences.

## Conclusions

To our knowledge, CRM1TR and CRM4TR represent the first tandem repeats derived entirely from single parental retrotransposons. The fact that these repeats arose independently from similar regions of two centromeric retrotransposon subfamilies and exhibit high sequence homology (97%-98%) to their extant parental retrotransposons suggests that the primary DNA sequence of these LTR/UTR regions have characteristics that favor illegitimate recombination and gene conversion. The discovery of tandem repeats originating from centromeric retrotransposons, some of which are enriched in CENH3 domains, raises the intriguing possibility that centromeric satellite repeats can be renewed or replaced by novel satellite repeats derived from retrotransposons belonging to the CR family.

## Methods

### Data mining

A BLASTable database was formatted from maize genome assembly RefGen_v2 [3]. CRM1TR and CRM4TR repeats were identified in RefGen_v2 based on their homology to LTR-UTR segment of fl-CRM1 and CRM4 elements using blastn. CRM1TR and CRM4TR monomers were extracted from RefGen_v2, using custom perl script, based on coordinates in the BLAST output file.

### Identification and dating of retrotransposon insertions

BLAST2seq and rpsblast was used to identify direct repeats and polyprotein domains of retrotransposons inserted between CRM-derived tandem repeats. Pairwise alignments of the 5^′^ and 3^′^ LTR of each full length retrotransposon with TSDs were generated using MUSCLE [61] and manually corrected using BioEdit [62]. The evolutionary distances between the 5^′^ and 3^′^ LTR pair of each retrotransposon (κ = estimated number of nucleotide substitutions per site) was estimated using K2P model in MEGA version 5 [63].

### Sequence analysis and SNP detection

Multiple sequence alignments of CRM1TR S and IR sequences were generated using MUSCLE [61] and visualized as well as manually edited using BIOEDIT [62]. SNPs were discovered using visual inspection of the multiple sequence alignment and scored as haplotypes in an excel sheet. Haplotype and monomer length graphs were generated using excel.

### Tandem repeat schematics

CRM1TR and CRM4TR schematics were generated using Fancygene [64] based on coordinates determined from multiple sequence alignments. Graphical visualization of CRM-derived tandem repeat loci in RefGen_v2 were created using JunctionViewer [65].

The dot plot was generated using the online server at http://www.vivo.colostate.edu/molkit/dnadot/.

## Abbreviations

BAC: Bacterial artificial chromosome; bp: base pairs; CENH3: Centromeric histone H3 variant; CentC: Centromeric tandem repeat of maize; ChIP: Chromatin immunoprecipitation; CR: Centromeric retrotransposon; CRM: Centromeric retrotransposon of maize; LTR: Long terminal repeat of a retrotransposon; Mb: Megabase; My: Million years; nt: Nucleotides; UTR: Untranslated region of a retrotransposon.

## Competing interests

The authors declare that they have no competing interests.

## Authors’ contributions

AS acquired the data. AS and GGP analyzed the data and wrote the manuscript. TKW and AS generated JunctionViewer images of CRM1TR and CRM4TR loci. All authors read and approved the final manuscript.

## Supplementary Material

Additional file 1 **Locus II CRM1TR monomer length is highly variable.** The number of full length CRM1TR monomers is plotted against 10 nt bins. Click here for file

Additional file 2 **Sequence similarity of CRM1TR consensus sequences with CRM1.** Multiple sequence alignment of consensus sequences derived from CRM1TR locus I monomers (LI_S-IR-E_B_) and locus II monomers (LII_S-IRx3-E_B_ and LII_S IRx3-E_A_) with the two CRM1 elements with three IRs (CRM1_B_ID1 and CRM1_B_ID2) and the consensus CRM1A (CRM1_A) and CRM1B (CRM1_B) sequences. Sequence similarities to CRM1A and CRM1B are indicated by green and yellow highlights, respectively. The horizontal arrows above the alignment indicate the CRM1TR subsequences S (red), IR (shades of gray), and E_A_/E_B_ (blue). Vertical black lines delineate the internal IR variants. Each IR sequence terminates in an A-rich region that is longer in monomers from locus I than those from locus II. LII_S-IRx3-E_A_ appears to be a recombinant with higher sequence similarity to CRM1A than CRM1B both in the A-rich region near, as well as downstream of, the IR-E_A_ junction. The recombination breakpoint is predicted to be on either end of CRM1A homologous region in CRM1TR_LII-IRx3-E_A_, where CRM1A and CRM1B sequences are indistinguishable (boxed with green highlight). Blue and red/orange stars on top of alignment indicate the position of SNPs in the S and IR regions respectively. Click here for file

Additional file 3 **CRM1TR S and IR haplotype from locus I and II are distinct.** Abundance of different haplotypes within CRM1TR S (top graph.) and IR (bottom graph) subsequence is graphed where percent of full length CRM1TR monomers sharing a given S or IR haplotype (listed along X-axis) is shown along Y-axis. No S or IR haplotypes are shared between CRM1TR locus I (L1) and II (L2). Numbers within brackets indicate the number of S and IR sequences used for haplotype analysis. Only three IR containing CRM1TR monomers are shown in the IR haplotype graph and these have been labeled c1, c2, c3 based on position from left to right. Click here for file

Additional file 4 **SNPs within S and IR subsequences from CRM1TR monomers at locus I and II.** Nucleotides at 7 positions (representing 7 SNPs) within ‘S’, and 8 positions (representing 7 SNPs and 1 in-del) within IR repeats of Fl-CRM1TR monomers from locus I (L1) (white background) and locus II (L2) (gray background) are shown. CRM1TR monomer names (IDs) are given in the leftmost column. Comparison of SNPs in the IR region from consensus sequences of locus I (L1_IR_consensus) and locus II (L2_IR*3_consensus) CRM1TR monomers and the two CRM1 elements (CRM1_ID1 and CRM1_ID2) with three IR copies (shown in yellow background) indicates that locus II monomers acquired three IR copies from existing CRM1B elements. Click here for file

Additional file 5 **Higher order structure within an internal repeat region of CRM4TR monomer.** a. Dot plot showing internal repeat in CRM4TR monomer. b. Multiple sequence alignment of 13 internal repeat units from CRM4TR consensus sequence shows relative sequence similarities between alternating units. Click here for file

Additional file 6 **Sequence of conserved CRM1B and CRM4B LTR motifs shown in Figure 1.** RB = recombination breakpoint of CRM1 recombinants described in [35]. Click here for file

Additional file 7 **CRM1TR and CRM4TR monomer termini map within a conserved UTR region of CRM1B and CRM4B.** The 251 nt region of CRM1B UTR that is homologous to a 263 nt region of CRM4B UTR is shown. The respective termini of CRM1TR and CRM4TR map (marked by red and blue stars respectively) near each other between two homologous domains of this region formed respectively by the first hundred nucleotides (blue shade) and last 143 nucleotides (pink shade). Click here for file

Additional file 8 **Fl-cDNAs map to CRM4TR repeats in CRM4TR containing BACs.** Tabular BLAST results showing that the three Fl-cDNAs (GenBank accessions BT019305.1, BT086630.1, and BT036284.1) map to the two overlapping CRM4TR-containing chr6 BACs AC213669 and AC186890 with a much higher bitscore than the next best hit, i.e. the chr1 BAC AC210216.3. Click here for file

Additional file 9 **Nucleotides surrounding the UTR-LTR junction of CRM1TR and CRM4TR repeats in full length consensus CRM1 and CRM4 sequences.** Short direct repeats required to create the UTR-LTR junction characteristic of CRM1TR and CRM4TR monomer junctions via illegitimate recombination in a single step are absent near the splice sites in CRM1B and CRM4B consensus sequences, thus the initial recombinants were likely generated in multiple steps, as illustrated in Figure 3a. (TIFF 88 kb) Click here for file
