# Supplementary figures and images for: Tandem repeats derived from centromeric retrotransposons
Source: BMC Genomics. 2013 Mar 4;14:142. doi: 10.1186/1471-2164-14-142 (PMC3648361; doi:10.1186/1471-2164-14-142)

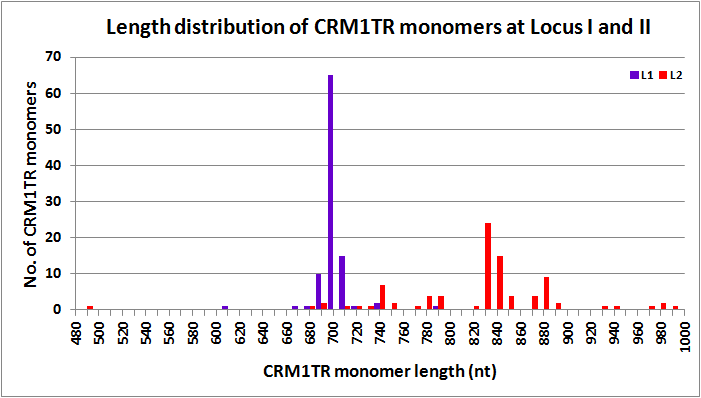

Supplement: Additional file 1 — Locus II CRM1TR monomer length is highly variable. The number of full length CRM1TR monomers is plotted against 10 nt bins. [file 1471-2164-14-142-S1.tiff]

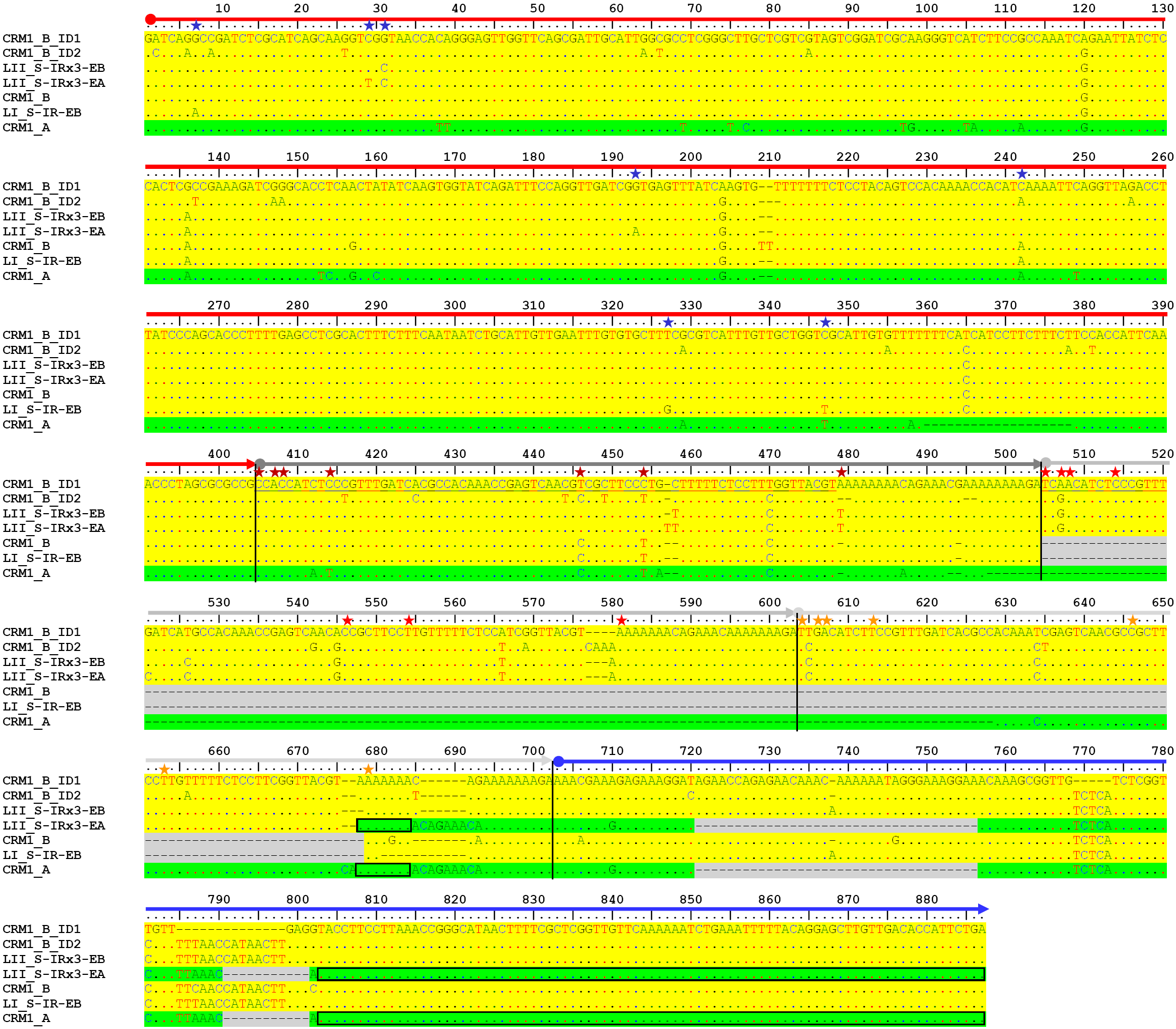

Supplement: Additional file 2 — Sequence similarity of CRM1TR consensus sequences with CRM1. Multiple sequence alignment of consensus sequences derived from CRM1TR locus I monomers (LI_S-IR-EB) and locus II monomers (LII_S-IRx3-EB and LII_S IRx3-EA) with the two CRM1 elements with three IRs (CRM1_B_ID1 and CRM1_B_ID2) and the consensus CRM1A (CRM1_A) and CRM1B (CRM1_B) sequences. Sequence similarities to CRM1A and CRM1B are indicated by green and yellow highlights, respectively. The horizontal arrows above the alignment indicate the CRM1TR subsequences S (red), IR (shades of gray), and EA/EB (blue). Vertical black lines delineate the internal IR variants. Each IR sequence terminates in an A-rich region that is longer in monomers from locus I than those from locus II. LII_S-IRx3-EA appears to be a recombinant with higher sequence similarity to CRM1A than CRM1B both in the A-rich region near, as well as downstream of, the IR-EA junction. The recombination breakpoint is predicted to be on either end of CRM1A homologous region in CRM1TR_LII-IRx3-EA, where CRM1A and CRM1B sequences are indistinguishable (boxed with green highlight). Blue and red/orange stars on top of alignment indicate the position of SNPs in the S and IR regions respectively. [file 1471-2164-14-142-S2.tiff]

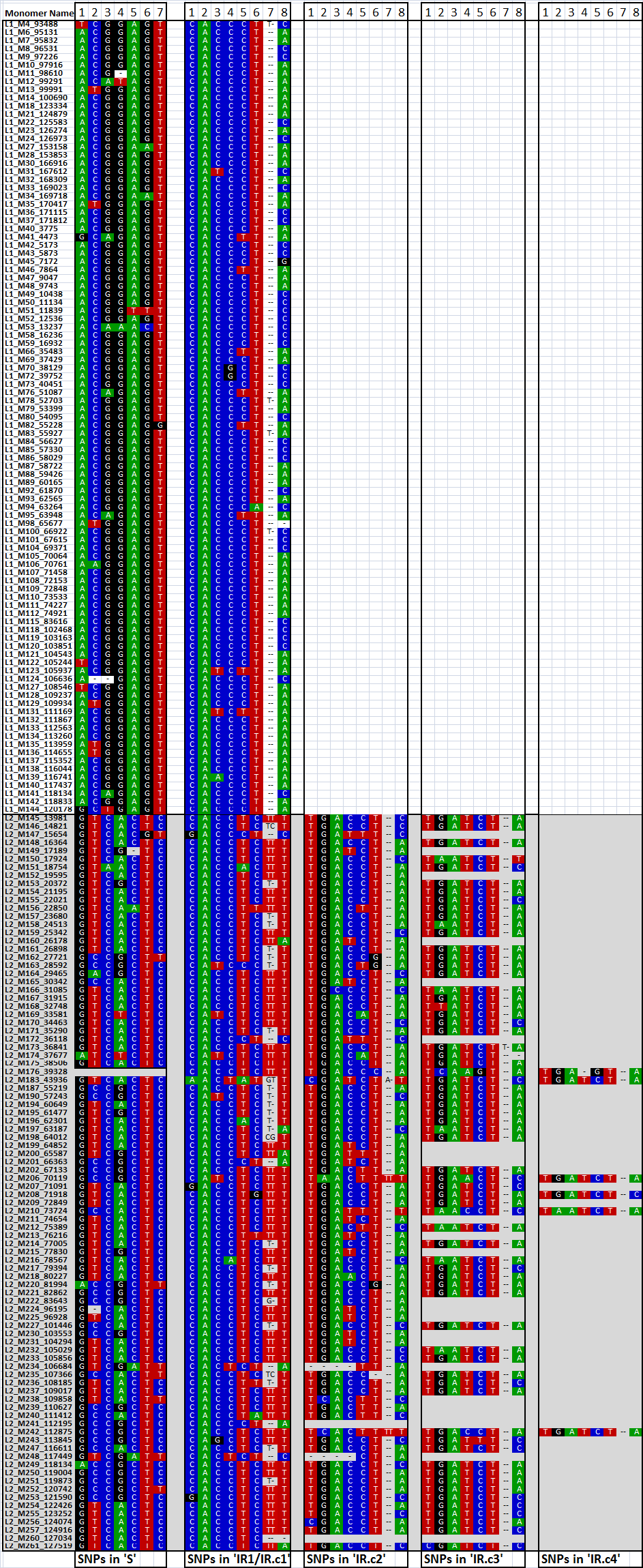

Supplement: Additional file 3 — CRM1TR S and IR haplotype from locus I and II are distinct. Abundance of different haplotypes within CRM1TR S (top graph.) and IR (bottom graph) subsequence is graphed where percent of full length CRM1TR monomers sharing a given S or IR haplotype (listed along X-axis) is shown along Y-axis. No S or IR haplotypes are shared between CRM1TR locus I (L1) and II (L2). Numbers within brackets indicate the number of S and IR sequences used for haplotype analysis. Only three IR containing CRM1TR monomers are shown in the IR haplotype graph and these have been labeled c1, c2, c3 based on position from left to right. [file 1471-2164-14-142-S3.tiff]

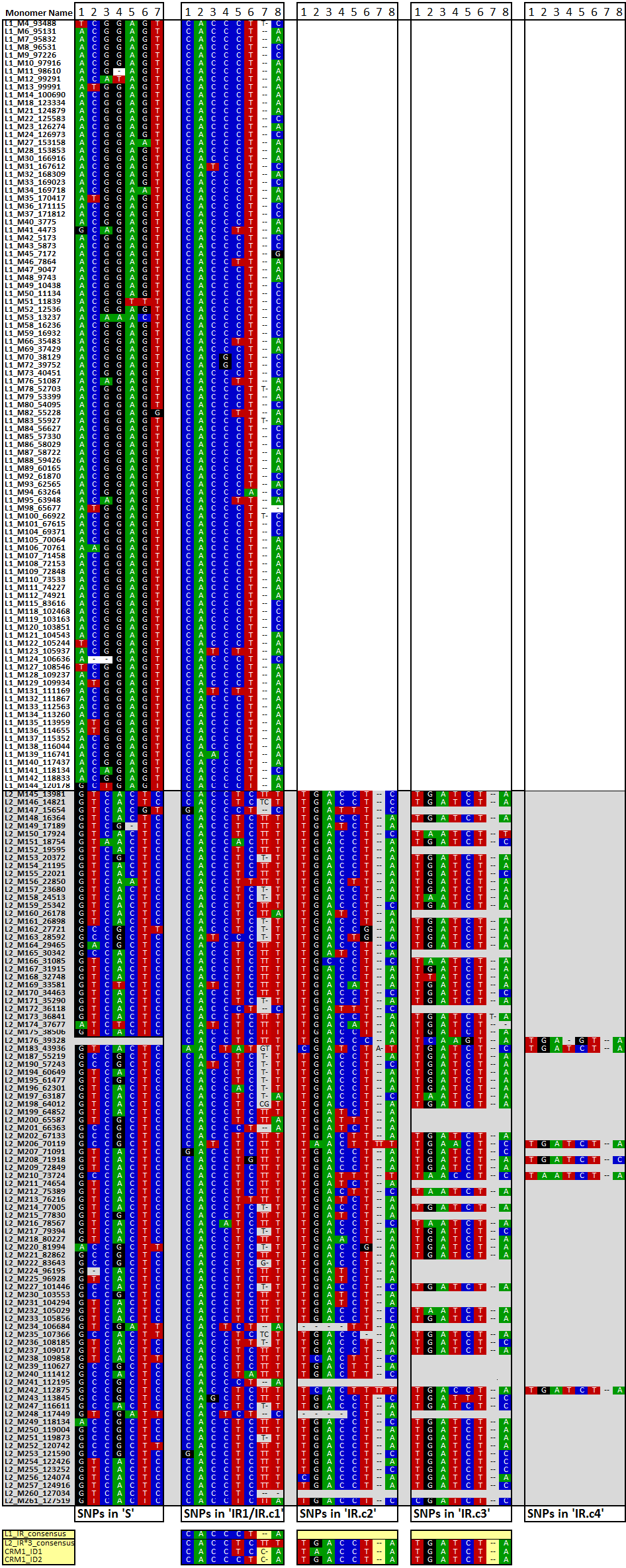

Supplement: Additional file 4 — SNPs within S and IR subsequences from CRM1TR monomers at locus I and II. Nucleotides at 7 positions (representing 7 SNPs) within ‘S’, and 8 positions (representing 7 SNPs and 1 in-del) within IR repeats of Fl-CRM1TR monomers from locus I (L1) (white background) and locus II (L2) (gray background) are shown. CRM1TR monomer names (IDs) are given in the leftmost column. Comparison of SNPs in the IR region from consensus sequences of locus I (L1_IR_consensus) and locus II (L2_IR*3_consensus) CRM1TR monomers and the two CRM1 elements (CRM1_ID1 and CRM1_ID2) with three IR copies (shown in yellow background) indicates that locus II monomers acquired three IR copies from existing CRM1B elements. [file 1471-2164-14-142-S4.tiff]

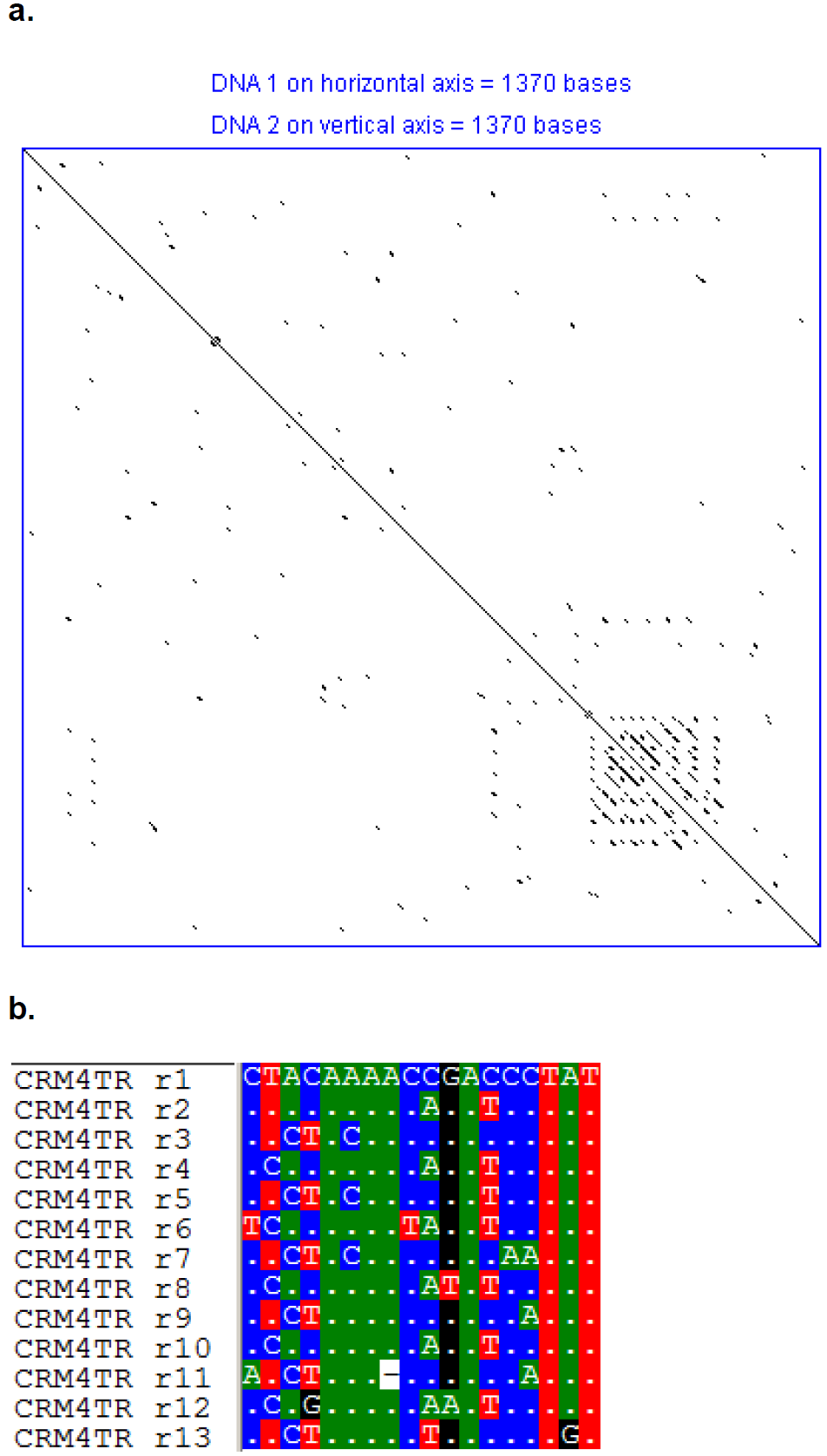

Supplement: Additional file 5 — Higher order structure within an internal repeat region of CRM4TR monomer. a. Dot plot showing internal repeat in CRM4TR monomer. b. Multiple sequence alignment of 13 internal repeat units from CRM4TR consensus sequence shows relative sequence similarities between alternating units. [file 1471-2164-14-142-S5.tiff]

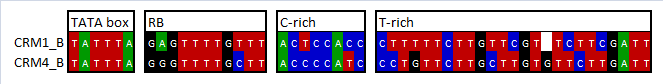

Supplement: Additional file 6 — Sequence of conserved CRM1B and CRM4B LTR motifs shown in Figure 1. RB = recombination breakpoint of CRM1 recombinants described in [35]. [file 1471-2164-14-142-S6.tiff]

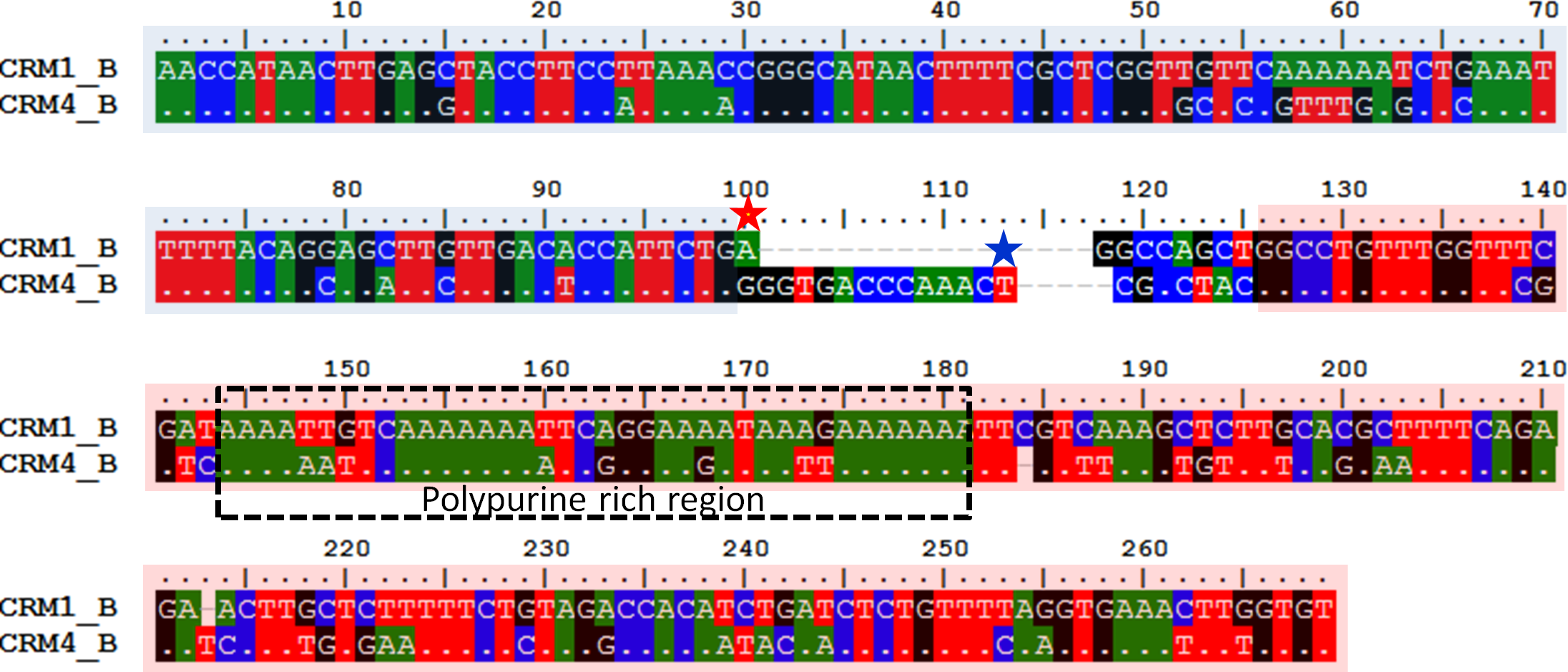

Supplement: Additional file 7 — CRM1TR and CRM4TR monomer termini map within a conserved UTR region of CRM1B and CRM4B. The 251 nt region of CRM1B UTR that is homologous to a 263 nt region of CRM4B UTR is shown. The respective termini of CRM1TR and CRM4TR map (marked by red and blue stars respectively) near each other between two homologous domains of this region formed respectively by the first hundred nucleotides (blue shade) and last 143 nucleotides (pink shade). [file 1471-2164-14-142-S7.tiff]

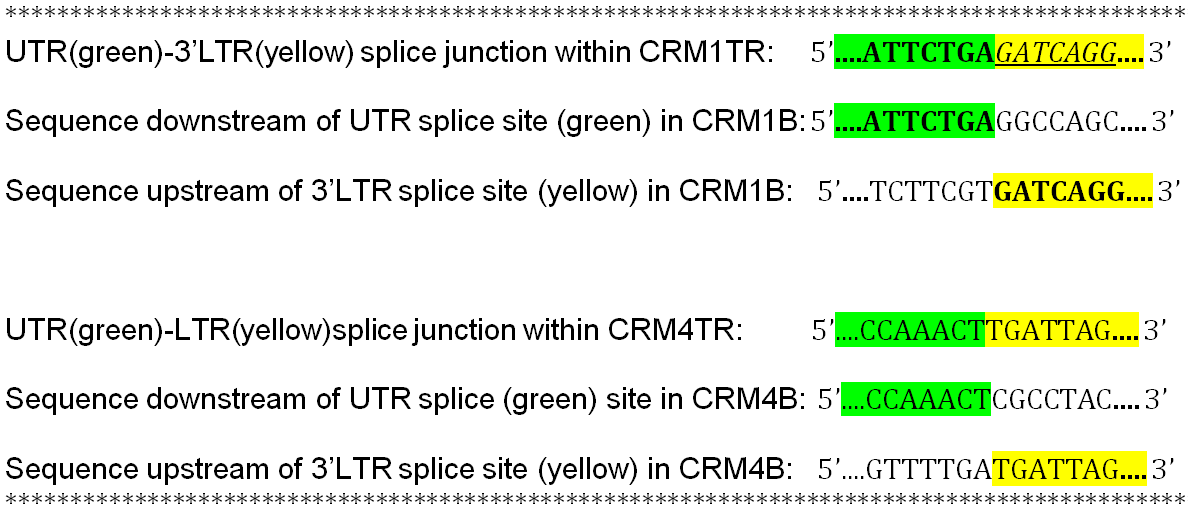

Supplement: Additional file 9 — Nucleotides surrounding the UTR-LTR junction of CRM1TR and CRM4TR repeats in full length consensus CRM1 and CRM4 sequences. Short direct repeats required to create the UTR-LTR junction characteristic of CRM1TR and CRM4TR monomer junctions via illegitimate recombination in a single step are absent near the splice sites in CRM1B and CRM4B consensus sequences, thus the initial recombinants were likely generated in multiple steps, as illustrated in Figure 3a. (TIFF 88 kb) [file 1471-2164-14-142-S9.tiff]
