# Supplementary material for: Tandem repeats derived from centromeric retrotransposons
Source: BMC Genomics. 2013 Mar 4;14:142. doi: 10.1186/1471-2164-14-142 (PMC3648361; doi:10.1186/1471-2164-14-142)
Supplement: Additional file 8 — Fl-cDNAs map to CRM4TR repeats in CRM4TR containing BACs. Tabular BLAST results showing that the three Fl-cDNAs (GenBank accessions BT019305.1, BT086630.1, and BT036284.1) map to the two overlapping CRM4TR-containing chr6 BACs AC213669 and AC186890 with a much higher bitscore than the next best hit, i.e. the chr1 BAC AC210216.3. [file 1471-2164-14-142-S8.docx]

## Additional file 8 –Fl-cDNAs map to CRM4TR repeats in CRM4TR containing BACs

| Query | Subject | % | Length | Mismatch | Gap | Query Start | Query End | Subject start | Subject end | Bitscore |
| --- | --- | --- | --- | --- | --- | --- | --- | --- | --- | --- |
| BT019305.1 | AC213669.4 | 99.04 | 519 | 5 | 0 | 22 | 540 | 126229 | 126747 | 913 |
|  | AC213669.4 | 100 | 346 | 0 | 0 | 536 | 881 | 114517 | 114862 | 625 |
|  | AC186890.3 | 99.04 | 519 | 5 | 0 | 22 | 540 | 32166 | 31648 | 913 |
|  | AC186890.3 | 100 | 346 | 0 | 0 | 536 | 881 | 31046 | 30701 | 625 |
|  | AC210216.3 | 95.42 | 524 | 19 | 1 | 22 | 540 | 87896 | 88419 | 838 |
|  | AC210216.3 | 97.69 | 347 | 7 | 1 | 536 | 881 | 87659 | 88005 | 587 |
| BT086630.1 | AC213669.4 | 100 | 518 | 0 | 0 | 172 | 689 | 70827 | 70310 | 935 |
|  | AC213669.4 | 100 | 139 | 0 | 0 | 38 | 176 | 71576 | 71438 | 251 |
|  | AC210216.3 | 96.33 | 518 | 19 | 0 | 172 | 689 | 81711 | 81194 | 848 |
|  | AC210216.3 | 97.12 | 139 | 4 | 0 | 38 | 176 | 87797 | 87659 | 233 |
| BT036284.1 | AC213669.4 | 100 | 524 | 0 | 0 | 320 | 843 | 77271 | 76748 | 946 |
|  | AC213669.4 | 100 | 324 | 0 | 0 | 1 | 324 | 107478 | 107801 | 585 |
|  | AC210216.3 | 97.33 | 524 | 14 | 0 | 320 | 843 | 88419 | 87896 | 883 |
|  | AC210216.3 | 97.23 | 325 | 8 | 1 | 1 | 324 | 87983 | 87659 | 542 |

Tabular BLAST results showing that the three Fl-cDNAs (GenBank accessions BT019305.1, BT086630.1, and BT036284.1) map to the two overlapping CRM4TR-containing chr6 BACs AC213669 and AC186890 with a much higher bitscore than the next best hit, i.e. the chr1 BAC AC210216.3.
